# Short-Wave Infrared Reflectance at 1050–1550 nm for Proximal Caries Detection: An In Vitro Diagnostic Accuracy Study Validated by Micro-CT

**DOI:** 10.3390/diagnostics16040548

**Published:** 2026-02-12

**Authors:** Friederike Litzenburger, Karl-Heinz Kunzelmann, Elias Walter, Falk Schwendicke, Katrin Heck

**Affiliations:** Department of Conservative Dentistry, Periodontology and Digital Dentistry, LMU University Hospital, LMU Munich, 80336 Munich, Germanyelias.walter@med.uni-muenchen.de (E.W.); falk.schwendicke@med.uni-muenchen.de (F.S.)

**Keywords:** dental enamel, image interpretation, computer-assisted, in vitro techniques, non-ionising radiation, sensitivity and specificity

## Abstract

**Background/Objectives:** Short-wave infrared reflectance (SWIRR) imaging is a non-ionising approach for proximal caries detection; however, the diagnostic impact of wavelength selection in reflectance imaging has not been systematically evaluated. **Methods:** This in vitro diagnostic accuracy study assessed SWIRR at 1050, 1200, 1300 and 1550 nm for proximal caries detection, using micro-computed tomography as the reference standard and digital bitewing radiography (BWR) as the clinical comparator. A total of 250 extracted permanent posterior teeth with sound or carious proximal surfaces were examined. SWIRR and BWR images were independently evaluated twice by two calibrated examiners using method-specific criteria. Diagnostic performance was quantified by sensitivity, specificity and accuracy; examiner reliability was analysed using kappa statistics, and pairwise comparisons were performed using McNemar tests with Holm–Bonferroni correction. **Results:** Diagnostic performance of SWIRR was wavelength dependent, showing increasing sensitivity and decreasing specificity with longer wavelengths. The highest overall accuracy was observed at 1050 nm (80.0%), exceeding that of BWR (73.8%) while maintaining comparable specificity and higher sensitivity. At 1550 nm, sensitivity was highest but accompanied by an increase in false-positive findings. **Conclusions:** SWIRR demonstrates high diagnostic potential for proximal caries detection, with 1050 nm providing the most favourable balance between diagnostic accuracy and specificity.

## 1. Introduction

Proximal caries detection using near-infrared light has gained increasing importance in clinical dentistry over the last few years. Theoretically, near-infrared light can be used with a range of wavelengths, while commercial solutions operate only with wavelengths of 780–850 nm. In previous studies, we demonstrated that near-infrared transillumination at 780 nm yielded high diagnostic accuracy for early proximal caries detection. However, near-infrared reflectance at 780 nm and 850 nm did not reach the diagnostic performance of the current standard of care, bitewing radiography (BWR), which has been reported to be highly accurate for the detection of cavitated and more advanced proximal lesions [1,2,3,4].

In transillumination, the tooth is positioned between the light source and the detector so that transmitted light is recorded; scattering within enamel and dentin generates contrast that depends on the local microstructure. In reflectance, the light source and detector are coaxial or side-by-side, and contrast arises from backscattered light from the dental tissues. Prior reports attributed lower-than-expected performance of near-infrared reflectance imaging to superficial reflection artefacts (e.g., specular highlights and surface defects) that reduce or mask lesion contrast [3,4]. Although this may appear counterintuitive given the artefact burden at 780–850 nm, fundamental optics and prior evidence suggest that reflectance at longer wavelengths (particularly in the short-wave infrared range; wavelengths above 1000 nm) should enhance lesion visibility.

From a materials perspective, enamel (predominantly inorganic) becomes more transparent with increasing wavelength because scattering decreases; transparency is particularly high around 1300 nm. By contrast, pronounced water-absorption bands near 1450 nm and 1940 nm reduce transparency again and strongly affect reflectance contrast [5]. These absorption effects are even more pronounced in dentin, which contains more organic matrix and water than enamel. Wavelengths above 1000 nm therefore represent a plausible range to improve the balance between lesion visibility and artefact susceptibility in reflectance imaging of enamel and dentin. Evidence remains limited. In a diagnostic study, Simon et al. reported improved reflectance imaging of proximal lesions when using wavelengths above 1500 nm [6]. However, there is a lack of systematic direct comparison of short-wave infrared reflectance (SWIRR) across discrete wavelengths (1050–1550 nm) with a radiological index test. This study therefore evaluated SWIRR at 1050, 1200, 1300 and 1550 nm for proximal caries detection using micro-computed tomography as the reference standard and digital BWR as the comparative index test. Given the reported properties of enamel and dentin, it can be assumed that the diagnostic accuracy of SWIRR increases with increasing wavelengths in the range between 1050 and 1550 nm.

In the present in vitro study, we aimed to investigate the diagnostic performance of SWIRR at 1050, 1200, 1300 and 1550 nm (index tests), respectively, for proximal caries detection. BWR was used as comparative test (given its role as standard of care), while micro-computed tomography (µCT) served as the reference standard. Our alternative hypothesis (H1) for this analysis was that the diagnostic performance of SWIRR increases with increasing wavelengths, while our null hypothesis (H0) was that no such association between performance and wavelength was present.

## 2. Materials and Methods

### 2.1. Sample Size

Based on previous work, near-infrared reflectance at 780 nm was assumed to yield a sensitivity for proximal caries detection of 30% [4]. In this study, we aimed to increase this sensitivity to 80% by increasing the short-wave infrared wavelengths. At an assumed power of 80%, an alpha of less than 0.05, and an assumed caries prevalence of 50%, the sample size resulted, using the primary outcome parameter of sensitivity, in a minimum of 28 samples. In our study design, one sample equals one tooth, which equals one proximal surface, because we deliberately evaluated only one proximal surface per tooth to avoid statistical dependencies. However, we substantially increased the sample size to 250 teeth (thus 250 proximal surfaces) for three specific reasons: (1) to ensure comparability with several previous diagnostic studies we and others have conducted with the same sample size, facilitating direct comparison of results; (2) to obtain more precise estimates of sensitivity, specificity, and overall accuracy with narrower 95% confidence intervals, which is important for clinical translation and evidence-based decision-making; and (3) to ensure adequate statistical power for secondary analyses including subgroup comparisons (e.g., molars vs. premolars) and assessment of inter-rater reliability [4,7,8,9]. This larger sample size provides robust evidence for the diagnostic performance of SWIRR across different wavelengths and tooth types.

### 2.2. Ethics Approval and Declarations

The study used fully anonymised extracted human teeth. The Ethics Committee at the Medical Faculty of LMU Munich confirmed that no ethics consultation/approval was required for this in vitro research project (project no. KB 20/019, date 26 October 2020). Teeth were extracted for clinical reasons and were provided to the investigators in pooled, fully anonymised form; no identifying patient data were collected or processed. For transparency, an earlier draft of this manuscript was posted as a preprint on Research Square [10].

### 2.3. Tooth Selection and Sample Preparation

Two hundred and fifty permanent human molars and premolars were selected from a pool of extracted teeth, without visible structural changes or damage other than proximal carious lesions. The teeth were free of any restorations and exhibited fully matured roots. From these 250 teeth, 250 proximal surfaces were evaluated (one proximal surface per tooth). Before µCT assessment, visual inspection of the proximal surfaces was performed directly under standardised lighting conditions according to the International Caries Detection and Assessment System (ICDAS) criteria (code 0 = sound, codes 1–5 = carious) for pre-stratification. It should be noted that this direct visualisation of proximal surfaces on extracted teeth represents a variation from the ICDAS application in a clinical context, where proximal surfaces are typically not directly visible due to proximal contact between adjacent teeth in vivo. A balanced sample of 125 sound and 125 carious proximal surfaces was assembled [11]. The carious samples consisted of 18 with an ICDAS score of 1, 69 with a score of 2, 25 with a score of 3, 4 with a score of 4, and 9 with a score of 5 [12]. Although the ICDAS classification of the surfaces only played a role in the pre-selection, all further comparisons were made according to the results of µCT evaluation. To ensure clinically relevant and comparable investigations and to select the most representative extent of caries, only proximal surfaces were included and evaluated that also had proximal contacts in vivo (exclusion of distal surfaces on wisdom teeth) and showed caries at a typical predilection site proximally at the level of the middle to lower third of the crown. Following an established and well-documented method, the samples were randomised into pairs and mounted in 3D-printed holders with proximal contact between them [4,7]. The teeth were stored in Ringer’s solution with 2% sodium azide at 4 °C and only removed briefly for the respective tests to prevent the samples from drying out.

### 2.4. Short-Wave Infrared Reflectance Imaging

Following the procedure used by Heck et al., 2021, a laboratory setup for SWIRR measurements was used on an optical breadboard [4]. The setup utilised cross-polarisation to minimise specular reflection artefacts: linear polarisers were positioned in the optical path with their polarisation axes oriented perpendicular to each other (90° offset). This cross-polarisation configuration effectively reduced surface glare while maintaining lesion contrast [13]. The components were adapted to specific wavelengths above 1000 nm. Four different light-emitting diodes were used as short-wave infrared light-sources at 1050, 1200, 1300 and 1550 nm (M1050L4, M1200L3, M1300L3 and M1550L3, Thorlabs, Newton, NJ, USA). All specimens were imaged in pairs with proximal contact established to maximise clinically relevant conditions. All 250 pairs of teeth were first imaged at one wavelength before the next wavelength was examined. This wavelength-complete imaging protocol ensured minimal air exposure time for each tooth and maintained consistent hydration conditions. The specimens were mounted on a stage using a magnet and then imaged occlusally. The test setup settings were consistent across all wavelengths and specimens, with no alterations made.

Images were taken with an indium gallium arsenide camera (WiDy Sens 320V-ST, resolution 320 × 256, New Imaging Technologies, Verrières-le-Buisson, France) using a 50 mm lens (LM50HC-SW, KOWA, Nagoya, Japan). The exposure time was 5 ms for the short-wave infrared wavelength of 1050 nm, 10 ms for 1200 and 1300 nm and 20 ms for 1550 nm [14]. All SWIRR images were acquired in a darkened room, and the equipment was kept in a black box to reduce stray light from the environment.

### 2.5. Digital Bitewing Radiography

To ensure that the grayscale distribution of the pixels corresponded to clinically obtained BWR, a validated in vitro model was used [7]. All specimens were radiographed without contact to the adjacent teeth to avoid overlaps [15]. The radiographs were obtained with a Heliodent DS Dental X-ray unit (Sirona, Bensheim, Germany, 60 kV, 7 mA, 200 mm focal-to-head distance cone, 0.08 s) and a digital charge-coupled device sensor (Intra-Oral II CCD sensor, Sirona, Bensheim, Germany; sensor size 30.9 × 1.0 × 7.0 mm).

### 2.6. Micro-Computed Tomography Scanning and Lesion Validation

High-resolution µCT served as the reference standard. All 250 teeth were scanned from the crown to the upper third of the root with a fully shielded cone-beam desktop µCT (µCT 40, Scanco Medical, Bassersdorf, Switzerland) at 70 kV and 114 µA, with the field of view restricted to 16.5 mm. Scans were acquired in standard resolution mode with an isotropic voxel size of 20 µm and a matrix size of 1000 × 1000 × 520 voxels. Each sample was reproducibly centred in a water-filled cuvette so that its orientation could be tracked during subsequent data analysis. System calibration was performed at 70 kV using a bone hydroxyapatite (BH) reference phantom with a density of 1200 mg HA/cm^3^. Image reconstruction yielded 3D datasets in ISQ format with 2048 grayscale levels. The plugin “KHKs_Scanco_ISQ_FileReader” was applied to import the data into Fiji [16] for the following image processing [17,18]. Analysis included all three spatial orientations (x, y, z) to determine lesion extent. Centreline-based segmentation was used to delineate enamel and dentin and to determine the centre of each structure. Centreline-based segmentation refers to a semi-automated approach in which the geometric centre of enamel and dentin volumes is computed to enable reproducible lesion depth assessment across all spatial planes. These segmentations were used as the reference for subsequent diagnostic comparisons [7].

### 2.7. Calibration and Training

Training and calibration for the evaluation of the findings of all index test methods (SWIRR), the comparative test method (BWR) and the reference test method (µCT) of both investigators (FL and KH) by a supervisor (KHK) included theoretical sessions on the basics of the scoring systems as well as calibration on 20 teeth that were not part of the sample size. Discordant diagnostic scorings were discussed, and a consensus finding was reached. After two weeks, the evaluation of the calibration set was repeated, and the inter- and intra-examiner reliability was then determined, achieving an agreement of more than 90%.

### 2.8. Evaluation

The evaluation of the findings of index test, comparative test and the reference test methods was conducted independently by both examiners (KH and FL). For each method, there were two evaluation cycles in random order with a two-week interval between the first and second run. After each evaluation cycle, all decisions of the two examiners were matched, different ratings were debated, and finally a consensus diagnosis was obtained. Surfaces that could not be clearly evaluated due to ambiguous findings or artefacts were marked as not assessable (na).

The µCT data were evaluated using segmentation and automatic centreline determinations for enamel and dentin as previously described by Heck et al. [7]. The µCT datasets were scrolled through all slices in all three directions to determine the deepest part of the lesion three-dimensionally and then scored as follows: 0—caries free, 1—caries in the outer half of the enamel, 2—caries in the inner half of the enamel, 3—caries in the outer half of the dentin, 4—caries in the inner half of the dentin.

The unaltered and complete images of BWR were scored according to Marthaler et al., with 0 for the absence of radiolucency, 1 and 2 for the presence of a radiolucency in the outer and inner halves of enamel and 3 and 4 for a radiolucency in the outer and inner halves of dentin [19].

SWIRR images were evaluated for the presence of a carious lesion (yes = 1/no = 0), analogous to Heck et al. and Litzenburger et al. [4,8]. A surface was considered diseased if a lighter area was detected in the proximal area within the sample.

All images from all examination methods were evaluated in a darkened room (window facing north, blinds two-thirds closed) on a calibrated monitor, compliant with DIN 6868–157. The seat distance was 60 cm (arm’s length), with at least 5 min of eye adaptation to the room environment before evaluation.

### 2.9. Statistics

For sample size calculation, SAS/STAT software (SAS/STAT, Version 15.1, Cary, NC, USA) using the Proc Power procedure was used. Statistical analysis was performed using the software SPSS (IBM SPSS Statistics for Windows, Version 27.0, Armonk, NY, USA) and Excel (Excel, Microsoft, Redmond, WA, USA).

Reliability assessments for the categorial variables BWR and µCT were calculated using linearly weighted Cohen’s kappa (wk), where a 1-category difference was considered less severe than a 2-category difference [20]. For the nominal (binary) variables SWIRR at 1050 to 1550 nm, Cohen’s kappa (k) was used (Table 1). All kappa values were interpreted according to Landis and Koch [21]. Where reported, 95% confidence intervals for k were derived from asymptotic standard error.

To compare the ratings of SWIRR and BWR with those of µCT (reference standard), contingency tables were generated (Table 2). For the statistical analysis in Table 3, which involves BWR and µCT data, enamel caries was defined according to scores 1 and 2, and dentin caries was defined according to scores 3 and 4. In addition, scores 1 to 4 were defined as carious lesions and score 0 as a sound surface. Not assessable ratings were pre-specified for exclusion from all analyses (listwise deletion). No imputation was performed.

The diagnostic performance of SWIRR and BWR was assessed using sensitivity (SE), specificity (SP) and overall accuracy (ACC). Each proportion is reported with 95% confidence intervals (CI) computed by the Clopper–Pearson exact method. In addition, we summarised positive and negative likelihood ratios (LR+, LR−) calculated from sensitivity and specificity; 95% CIs for LRs were obtained using the log-method (normal approximation on ln(LR)). Because SWIRR ratings were binary (lesion yes/no) and no threshold variation was available, receiver operating characteristic (ROC) analysis and area under the curve (AUC) were not computed. Overall accuracy was calculated as the number of correctly classified surfaces divided by the total number of reference surfaces, expressed as a percentage. Likewise, overestimation was calculated as the number of false-positive ratings divided by the number of reference sides, and underestimation was calculated as the number of false-negative values divided by the number of reference sides (Table 3).

Paired comparisons of BWR with each SWIRR wavelength on the same tooth surfaces used exact McNemar’s test (two-sided) for sensitivity (restricted to µCT-positive), specificity (restricted to µCT-negative), and accuracy (correct vs. incorrect relative to µCT); for all McNemar analyses, we report the exact two-sided *p*-value (binomial test). Holm–Bonferroni corrections were applied to control multiple testing within outcome families (four comparisons: 1050/1200/1300/1550 nm vs. BWR), maintaining a family-wise α = 0.05. Unless otherwise stated, tests were two-sided with a significance level of α = 0.05.

## 3. Results

Almost perfect agreement for all methods was observed in the intra- and inter-examiner reliability assessment (Table 1) [21].

**Figure 1:**
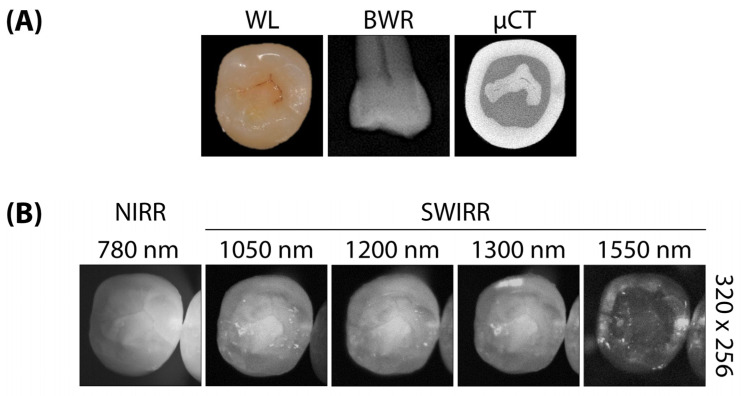
A permanent premolar with a proximal enamel caries photographed with white light (WL) in occlusal view, imaged in a cropped bitewing radiograph (BWR) and in cross-section with micro-computed tomography (µCT) (**A**). Corresponding short-wave infrared reflectance (SWIRR) images at 1050, 1200, 1300, 1550 nm and 780 nm near-infrared reflectance (NIRR) images (**B**). A 780 nm near-infrared reflectance (NIRR) image is shown for illustrative comparison only and was not included in the study dataset or statistical analyses.

**Figure 2:**
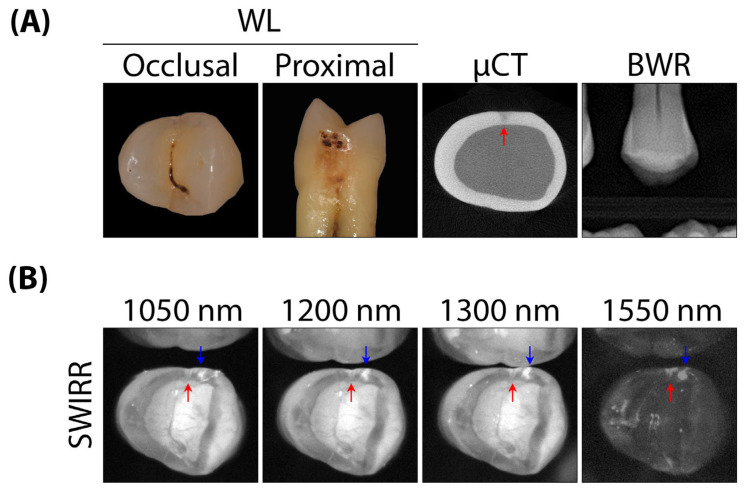
White light (WL) image from occlusal view, without any signs of caries and with almost undetectable chipping in the enamel. The premolar is imaged using WL, micro-computed tomography (µCT) and a cropped version of a bitewing radiograph (BWR) (**A**). In short-wave infrared reflectance (SWIRR), the chipping (blue arrow) increasingly risks false-positive interpretation as wavelength increases from 1050 to 1550 nm due to the enhancement of contrast, whereas the visibility of the caries lesion remains comparatively stable (red arrow) (**B**).

**Figure 3:**
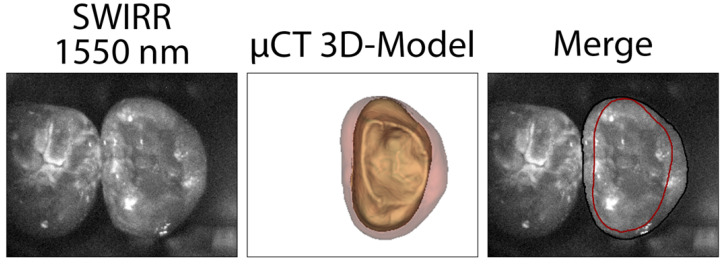
Short-wave infrared reflectance (SWIRR) image at 1550 nm with an indistinct enamel-dentin junction (EDJ). By segmenting the microtomographic data of the sample (µCT 3D-Model) and merging the 3D model with the SWIRR image, the “true” EDJ can be mapped (red line).

The presence of proximal caries was assessed using a yes/no decision, as the actual interface between enamel and dentin could not be reliably defined using SWIRR, particularly at 1550 nm (Figure 1, Figure 2 and Figure 3). Differentiation of the enamel-dentin interface was possible in approximately 80% of the 250 proximal surfaces at 1050 nm. However, this percentage decreased with increasing wavelength, falling to 77% at 1200 nm, 67% at 1300 nm, and 57% at 1550 nm.

Depending on the method used, the following proportions of the 250 surfaces were classified as carious: 50% with ICDAS, 12.4% with BWR, 20.4% with SWIRR at 1050 nm, 28.8% with SWIRR at 1200 nm, 30.8% with SWIRR at 1300 nm and 48.8% with SWIRR at 1550 nm (Table 2). The overall accuracy was 80% for SWIRR 1050 nm, 77% for 1200 nm, 78% for 1300 nm, 72% for 1550 nm and 74% for BWR. With increasing wavelengths, SWIRR revealed increasing sensitivity and decreasing specificity for proximal carious lesions. SWIRR at 1550 nm demonstrated the highest sensitivity and the lowest specificity for caries and other non-caries-related changes in the dental tissue (Table 3). Subjectively, 1200 and 1300 nm provided high transparency of the enamel, and 1050 nm exhibited minimal reflection artefacts (Figure 1). BWR showed significantly lower sensitivity than SWIRR across wavelengths (Holm-adjusted *p* < 0.05), and the highest specificity (0.99; specificity at 1050 nm was comparable at 0.98) (Figure 1). Using SWIRR at 1050–1550 nm, the tendency to overestimate caries increased with the wavelength, while the underestimation decreased (Table 2).

After Holm–Bonferroni correction, SWIRR 1050–1550 nm was higher than BWR in terms of sensitivity (all adj. *p* < 0.05; raw *p*: 1050 nm = 0.001, 1200/1300/1550 nm *p* < 0.001). Specificity was lower than BWR for 1200/1300/1550 nm (adj. *p* < 0.05; raw *p* < 0.001), with no difference for 1050 nm (raw *p* = 0.375). Accuracy showed no significant differences after multiplicity adjustment (raw *p*: 1050 nm = 0.017; 1200 nm = 0.350; 1300 nm = 0.200; 1550 nm = 0.762). Pre-specified not assessable ratings were excluded by listwise deletion. Overall, 2/250 surfaces (0.8%) were not assessable in BWR, and 0/250 in SWIRR (Table 2).

## 4. Discussion

This in vitro study investigated the diagnostic performance of proximal caries detection using SWIRR at wavelengths of 1050, 1200, 1300 and 1550 nm on permanent posterior teeth. Under optimised laboratory conditions, images with optimum exposure, high depth of field, and minimised reflection artefacts were obtained. BWR served as the comparator and µCT as the reference standard. There have been previous studies focusing on the validity of SWIRR in proximal caries detection [6,22,23]. These studies provide important information on the diagnostic performance of SWIRR for the detection of caries, although they were conducted on premolars only. In these studies, SWIRR was used above 1500 nm, as it was assumed that caries would be visualised with greater contrast in this range of wavelengths due to the increase in water absorption. To date, it has not been clarified whether it must be this range of wavelengths, which starts at 1500 nm, or if a lower range between 1000 and 1500 nm can provide comparable or higher diagnostic accuracy. There is a lack of in vitro studies with a broad sample pool that includes molars and premolars and a balanced and clinically realistic distribution of caries. This analysis is therefore of high clinical relevance, as the understanding of the diagnostic strengths and limitations of SWIRR across different wavelengths provides a scientific basis for the development and optimisation of future diagnostic devices.

Compared to BWR, which showed limited sensitivity, especially for enamel lesions, SWIRR at 1050–1300 nm offered a higher detection rate for early caries. These findings are consistent with previous studies highlighting the limitations of radiographic methods in detecting initial lesions that may lack sufficient mineral loss to be radiographically visible [24]. In our study, the sensitivity for enamel lesions using BWR was only 12%, which is lower than in many earlier reports. This discrepancy can be explained by the composition of our sample, which was deliberately designed to reflect a clinically realistic distribution of caries stages. Many of the early lesions were confined to or located near the outer enamel layer and did not exhibit the degree of demineralisation required for radiographic visualisation. Moreover, the present investigation aimed to assess diagnostic detectability, not treatment planning. Therefore, small and incipient lesions—although potentially not treated invasively—were included and considered diagnostically relevant. This approach highlights the clinical value of detection tools that are sensitive even in the early stages of lesion development.

To establish a valid diagnostic reference, all crowns of the teeth were scanned in full volume using high-resolution micro-computed tomography (µCT). Three-dimensional datasets were reconstructed and analysed in all spatial planes (x, y, z) to identify lesion extension and morphology. Enamel and dentin were segmented digitally, allowing for the visualisation of demineralised volumes and their spatial relationship to anatomical landmarks such as the enamel-dentin junction [7]. This volumetric approach provides a comprehensive and objective assessment of lesion depth and avoids oversimplified assumptions of linear progression from surface to pulp. Three-dimensional µCT evaluation is widely regarded as the gold standard in in vitro caries research, as it enables reproducible and high-resolution lesion characterisation across a range of stages.

In contrast to previous reports suggesting optimal caries contrast above 1500 nm due to increased water absorption [6,13], our findings revealed that SWIRR at 1050 nm yielded the highest diagnostic accuracy. At this wavelength, high specificity and reduced surface-related artefacts were observed, making it particularly suitable for detecting early proximal lesions without ionising radiation. With increasing wavelengths, sensitivity improved, but specificity declined. At 1550 nm, although the highest sensitivity was achieved, the frequency of false positives significantly increased. These findings underline the trade-off between sensitivity and specificity when selecting optimal wavelengths for SWIRR imaging.

In the SWIRR images, enamel and dentin show markedly different optical properties depending on the wavelength (Figure 1 and Figure 2). The physical principles are important for the effect of different wavelengths on reflectance imaging: The attenuation of light in healthy enamel is mainly based on Rayleigh scattering, but also partly on absorption by water, which is present in the enamel at about 12% by volume [25,26]. The scattering decreases with increasing wavelengths, reaching its highest transparency at 1300 nm [27]. The light directed onto the tooth from the occlusal side shines through the enamel without significant attenuation, so that the tissue appears rather dark in the reflectance. Above 1300 nm, an absorption band of water begins, and the transparency of the enamel decreases slightly. In healthy dentin, the strong attenuation of the light is primarily due to the scattering and absorption by water, which is present in dentin at approximately 25% by volume [25,26,28,29]. The backscattering depends on the scattering-absorption ratio. For reflectance imaging with near-infrared wavelengths, such as 780 nm, the scattering in enamel as well as the backscattering in dentin are generally high. Therefore, the whole image of the tooth appears brighter and the differentiation between enamel and dentin is often unclear (Figure 1). At short-wave infrared wavelengths, the dentin appears darker with increasing wavelengths. This has been previously described by Zakian et al. and confirmed by recent investigations [30,31]. The images at 1550 nm appeared overall very dark (Figure 1, Figure 2 and Figure 3). This was reflected in the image appearance: while SWIRR at 1550 nm provided the highest sensitivity, it also showed a markedly increased rate of false-positive findings, likely due to enhanced backscattering from superficial irregularities and structural artefacts. In contrast, SWIRR at 1050 nm provided a favourable balance of sensitivity and specificity, minimal reflection artefacts, and clearly distinguishable contrast between enamel and dentin.

In our qualitative analysis, the visibility of the enamel-dentin junction (EDJ) varied by wavelength and was most prominent at 1050 nm. At 1050 nm, enamel and dentin were most clearly visually distinguishable and the EDJ was detectable in 80% of the 250 proximal surfaces. This value decreased with increasing wavelength and was lowest at 1550 nm (57%). In comparison, previous studies on reflectance imaging with wavelengths at 780 and 850 nm reported only 22 and 47% visibility of the interface between enamel and dentin [3,4]. However, due to the natural curvature of the EDJ and the oblique trajectory of dentinal tubules, the interface depicted in 2D reflectance images may not reliably correspond to the anatomical EDJ. Whether the demarcation between enamel and dentin in the two-dimensional reflectance images reflects the actual EDJ has not been validated in any of the studies available to date. Figure 3 presents an approach to the problem. It shows an image of the segmented µCT data merged with the corresponding SWIRR image, in this case at 1550 nm. The trivial one-to-one projection of the EDJ in SWIRR images obviously does not always match the actual EDJ, as shown here using the µCT data (Figure 3). It can be found that the two-dimensional depiction of the EDJ using SWIRR depends on the angle of light incidence. If the tooth is photographed with the enamel-dentin interface perpendicular to the sensor, the enamel and dentin appear exactly separated from each other. This is more often the case with premolars than with molars. In other examples, the light hits the axis between enamel and dentin at an angle and the interface is less clearly visible. The merging of the three-dimensional µCT model and the corresponding SWIRR image demonstrates that the depicted interface between enamel and dentin in two-dimensional SWIRR findings is not always congruent with the “true” EDJ (Figure 3). Given these anatomical and optical constraints, we chose a dichotomous classification system for SWIRR (caries present/absent) rather than attempting to stage lesion depth, which is warranted given the limited geometric fidelity of the EDJ in 2D reflectance images. This approach proved to be robust and clinically meaningful in detecting early carious changes, particularly in enamel. SWIRR may therefore serve as a valuable non-ionising diagnostic adjunct, especially in contexts requiring repeated monitoring, such as in paediatric patients or individuals at high caries risk.

The increasing rate of false positive findings at higher wavelengths is likely to translate into overestimation and overtreatment. These false-positive SWIRR findings at higher wavelengths were mainly due to artefacts that led to misinterpretation: Superficial damages such as chipping, abraded facets or calculus led to increased backscattering (Figure 2). The superficial defects revealed a greater backscattering of light than deeper lesions. In comparison to near-infrared reflectance imaging at 780 and 850 nm, SWIRR between 1050 and 1300 nm exhibited higher diagnostic validity [1,4]. Even when observing the SWIRR images without statistical analysis, the information content of the findings between 1050 and 1300 nm is higher than that of the images at 780 nm (Figure 1).

This study has certain limitations. As an in vitro investigation, it does not fully replicate the clinical setting, where factors such as saliva, adjacent teeth, soft tissues, and patient movement may influence image quality and accessibility. Moreover, although artificial spacing allowed for standardised imaging, the absence of natural contact areas may have affected lesion morphology and visualisation. Future in vivo studies should evaluate SWIRR imaging under clinical conditions and investigate its diagnostic value in different caries risk groups. In addition, the potential of multi-wavelength or hyperspectral reflectance imaging warrants further exploration to refine diagnostic capabilities. A promising approach could be the analysis of contrast differences across multiple wavelengths at identical illumination angles. Since penetration depth and scattering behaviour vary with wavelength, the observed reflectance from enamel-dentin junctions or carious lesions may shift accordingly [32]. In our study, the EDJ appeared visible in a large proportion of cases at 1050 to 1300 nm. This observation raises the hypothesis that wavelength-dependent reflectance patterns may provide indirect information about lesion depth and location. Measuring such contrast shifts systematically could help to approximate the true position of the EDJ and potentially improve the depth resolution of SWIRR imaging in future developments. Furthermore, integration of artificial intelligence (AI) and deep learning algorithms represents a promising avenue for enhancing diagnostic accuracy and clinical applicability of SWIRR imaging. Recent systematic reviews have demonstrated that deep learning models achieve high diagnostic accuracy in caries detection from radiographic and photographic images [33,34,35,36]. AI-assisted analysis could potentially automate lesion detection, differentiate lesion severity, and reduce inter-examiner variability in SWIRR image interpretation. Future research should explore the development of AI-based diagnostic tools specifically trained on SWIRR images at different wavelengths, which could facilitate standardised clinical implementation and real-time decision support for practitioners.

The initial hypothesis that longer SWIRR wavelengths would result in superior diagnostic performance for proximal caries detection must be rejected. Diagnostic accuracy did not increase continuously with wavelength. Rather, the findings demonstrate that each wavelength range has specific advantages and limitations. The best overall diagnostic conditions were observed at 1050 nm, where a favourable balance between sensitivity, specificity and artefact minimisation was observed. In addition, the subjective impression of the strongest contrast between sound and demineralised tissues at 1050 nm supports this conclusion. This contradicts earlier assumptions that maximum caries contrast would occur at 1550 nm due to increased water absorption. However, further validation is needed to refine this impression. Future studies combining SWIRR imaging at different wavelengths with high-resolution three-dimensional µCT datasets may provide additional insight. The spatial merging of SWIRR images with µCT-based lesion models could support the development of reflectance-based staging systems and help define caries severity more precisely. In this context, a wavelength-dependent contrast analysis may enable an improved approximation of the true enamel-dentin junction and lesion extension.

## 5. Conclusions

Short-wave infrared reflectance has a high potential for proximal caries detection. At 1050 nm, SWIRR showed the highest accuracy and a lower tendency to overestimate caries than at 1200–1550 nm; the difference versus BWR did not remain statistically significant after Holm–Bonferroni adjustment. The use of light with a wavelength longer than 1050 nm improves the sensitivity but reduces the specificity of the SWIRR. A multi-wave combination for SWIRR could balance the individual advantages and disadvantages between absorption and reflectance of the dental tissues. As an alternative to BWR, SWIRR at 1050 nm demonstrated the highest diagnostic potential and proved to be a clinically relevant non-ionising option for the detection of approximal caries.

## Figures and Tables

**Table 1:** Inter- and intra-examiner reliability using Cohen’s kappa values for dichotomous evaluation of short-wave infrared reflectance at all investigated wavelengths and linearly weighted κ-values for nominal evaluation of BWR, with corresponding 95% confidence intervals in parentheses.

		Inter-	Intra-	
	WL	Examiner 1 vs. 2	Examiner 1	Examiner 2
SWIRR	1050 nm	1.00	0.96	1.00
		(1.00–1.00)	(0.92–1.00)	(1.00–1.00)
	1200 nm	0.90	0.94	0.94
		(0.84–0.96)	(0.89–0.99)	(0.89–0.99)
	1300 nm	0.93	0.96	0.96
		(0.89–0.98)	(0.93–1.00)	(0.92–1.00)
	1550 nm	0.94	0.88	0.91
		(0.90–0.99)	(0.82–0.94)	(0.86–0.96)
BWR		0.86	0.97	0.85
		(0.78–0.94)	(0.94–1.00)	(0.76–0.93)

BWR—bitewing radiographs, SWIRR—short-wave infrared reflectance, WL—wavelengths.

**Table 2:** Cross table of digital bitewing radiography and short-wave infrared reflectance at wavelengths from 1050 to 1550 nm, crossed with the corresponding results of micro-computed tomography.

		BWR						SWIRR								
								1050 nm		1200 nm		1300 nm		1550 nm		
		0	1	2	3	4	Na	0	1	0	1	0	1	0	1	Total
µCT	0	155	0	1	0	0	1	153	4	139	18	138	19	108	49	157
	1	17	0	0	1	0	0	15	3	12	6	12	6	6	12	18
	2	16	4	1	2	0	0	12	11	11	12	12	11	8	15	23
	3	31	3	6	6	0	1	18	29	16	31	11	36	6	41	47
	4	0	1	0	1	3	0	1	4	0	5	0	5	0	5	5
	Total	219	8	8	10	3	2	199	51	178	72	173	77	128	122	250

BWR—bitewing radiograph, Na—not assessable, SWIRR—short-wave infrared reflectance, µCT—micro-computed tomography.

**Table 3:** Sensitivity, specificity, positive Likelihood Ratio, negative Likelihood Ratio, and accuracy for the following test methods and modalities: short-wave infrared reflectance at wavelengths of 1050, 1200, 1300 and 1550 nm, and digital bitewing radiography. Micro-computed tomography served as the reference test, with 95% confidence intervals in parentheses.

		WL	Sensitivity	Specificity	LR+	LR−	ACC
SWIRR	Carious lesion	1050 nm	0.51	0.98	19.84	0.51	0.80
			(0.40–0.61)	(0.94–0.99)	(7.38–53.28)	(0.41–0.62)	(0.75–0.85)
		1200 nm	0.58	0.89	5.06	0.47	0.77
			(0.47–0.68)	(0.82–0.93)	(3.17–8.08)	(0.37–0.61)	(0.71–0.82)
		1300 nm	0.62	0.88	5.15	0.43	0.78
			(0.52–0.72)	(0.82–0.93)	(3.29–8.08)	(0.33–0.56)	(0.73–0.83)
		1550 nm	0.79	0.69	2.52	0.31	0.72
			(0.69–0.86)	(0.61–0.76)	(1.95–3.25)	(0.21–0.47)	(0.66–0.78)
BWR	Carious lesion		0.30	0.99	47.48	0.70	0.74
			(0.21–0.41)	(0.96–1.00)	(6.57–343.18)	(0.61–0.80)	(0.68–0.79)

ACC—accuracy, BWR—bitewing radiograph, LR+—positive Likelihood Ratio, LR−—negative Likelihood Ratio, SWIRR—short-wave infrared reflectance, WL—wavelength, µCT—micro-computed tomography. Sensitivity and specificity were calculated against the µCT reference on µCT-positive and µCT-negative surfaces, respectively. Accuracy refers to the proportion correctly classified among all surfaces; 95% CIs for proportions use exact Clopper–Pearson intervals (bounded within [0, 1]); likelihood-ratio CIs use the log method and are strictly positive. Paired comparisons of SWIRR wavelengths with BWR used exact McNemar tests; multiplicity controlled with Holm–Bonferroni within each outcome family (FWER α = 0.05).

## Data Availability

The raw data required to reproduce the above findings are available to download from: https://doi.org/10.5282/ubm/data.444 (accessed on 1 January 2026), https://doi.org/10.5282/ubm/data.445 (accessed on 1 January 2026), https://doi.org/10.5282/ubm/data.446 (accessed on 1 January 2026) and https://doi.org/10.5282/ubm/data.447 (accessed on 1 January 2026).
